# The MOGA multi-modal framework based on graph augmentation networks for drug response prediction

**DOI:** 10.1016/j.isci.2026.115167

**Published:** 2026-03-11

**Authors:** Kaiyuan Zhang, Runze Wang, Tianyi Zang, Yanli Zhao

**Affiliations:** 1 Faculty of Computing, Harbin Institute of Technology, Harbin, Heilongjiang 150001, China; 2 Medical College, Qinghai University, Xining, Qinghai 810016, China

**Keywords:** Artificial intelligence, Bioinformatics, Drug dispensing, Omics, Precision medicine

## Abstract

Personalized cancer treatment faces challenges due to tumor heterogeneity and limitations in existing computational methods regarding multi-omics integration. To address these limitations, this study presents MOGA, an advanced framework for drug response prediction. MOGA constructs a heterogeneous graph integrating cell line multi-omics, drug chemical structures, and response data, utilizing a relational graph convolutional network (RGCN) to model complex interactions. Crucially, a type-specific graph augmentation strategy is proposed, which categorizes neighbor influence to preserve critical sensitive signals while reducing noise from non-sensitive nodes. Experimental results demonstrate that MOGA significantly outperforms competitive baselines in both AUROC and AUPR. Ablation studies and case analysis confirm that the integration of multi-omics data and the targeted augmentation mechanism are central to these performance gains, validating MOGA’s potential for precision oncology.

## Highlights

• Directed RGCN distinguishes sensitive/resistant edges to capture biological directionality
• Six dedicated encoders capture modality-specific features to minimize omics information loss
• Type-specific augmentation perturbs non-sensitive nodes to reduce noise and preserve signals
• Outperforms baselines on GDSC/CCLE and generalizes robustly to clinical TCGA datasets

## Graphical abstract

**Figure:**
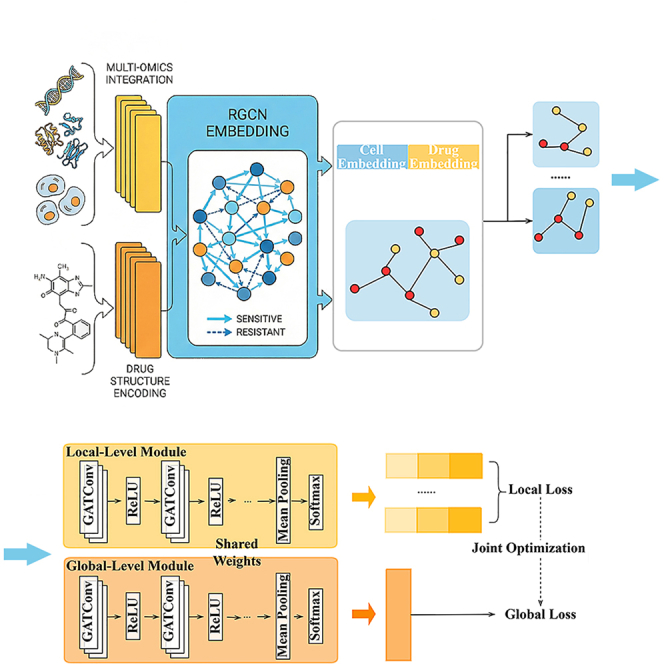

## Introduction

Drug response prediction (DRP) is an important research challenge in personalized medicine and drug development and is of great significance for improving treatment effects and reducing adverse reactions. Personalized medicine aims to find the most suitable treatment for each patient. Especially in cancer treatment, due to the heterogeneity of patient tumors, individuals with the same type of cancer may respond differently to the same therapy.^1^ It is crucial to accurately predict each patient’s response to drugs. However, DRP faces many challenges, including the complex interactions between drug compounds and cellular entities. Traditional methods have difficulty capturing these complex networks, resulting in suboptimal prediction results and limited interpretability. In addition, current DRP methods also face challenges such as data heterogeneity, limited sample size, and the need for multi-omics integration.^2^ For example, existing clinical patient databases lack sufficient drug response data for effective training, while drug screening data based on large-scale cell lines are available, but there is data heterogeneity and the need for multi-omics integration.^3^ In drug development, the interaction between genetic diversity and environmental influences brings uncertainty to DRP, and the dynamic level of gene expression also increases the variability of drug response.^4^ In recent years, with the development of artificial intelligence technology, artificial intelligence has been increasingly used in predicting the potential response of cancer patients to drugs. Despite this, the currently available clinical data still have limitations, and computational models mostly rely on large-scale cell line drug screening data for training. Fortunately, the scientific community has provided a large number of pharmacogenomic data resources, such as Genomics of Cancer Drug Sensitivity (GDSC),^5^ Cancer Cell Line Encyclopedia (CCLE),^6^ Cancer Therapy Response Portal (CTRP),^7^ and The Cancer Genome Atlas (TCGA) program.^8^ These projects not only provide a large number of molecular spectrum data of cancer cell lines, including somatic mutations, copy number aberrations, proteomic data, etc., but also contain data on the response of these cell lines to various targeted therapies and chemotherapy, providing researchers with valuable resources and accelerating the research progress of personalized cancer treatment.^9^^,^^10^

Based on preclinical and clinical drug response data and gene maps, many studies have used machine learning and deep learning algorithms to build cancer drug response (CDR) prediction models.^11^ These models are divided into multi-drug models and single-drug models according to the number of drugs used for training.^12^ In the clinical CDR prediction task, the single-drug model is to build a model for each drug based on omics data alone.^13^ This type of traditional prediction method has the disadvantages of high resource consumption, high cost, and long processing time. In recent years, with the rapid development of pharmacogenomics, a large amount of genomic data have been generated, covering gene mutations, copy number variations, gene expression profiles, etc., as well as many large datasets on drug sensitivity and resistance of different cancer cell lines, which have promoted the development of computational models for DRP based on multi-omics.^14^^,^^15^ In this context, the integration of bioinformatics and machine learning has opened up a new research path for DRP. Previously, a series of models, including ridge regression,^16^ support vector machines,^17^ and transfer learning,^18^ have been developed to quantify drug responses based on half-maximal inhibitory concentration (IC50) values using existing data, where a low IC50 value indicates that a sensitive drug may have a significant response to a specific target cell line, and vice versa, indicating that the drug-response relationship is not significant.^19^ Although these methods have achieved certain results, most of them lack comprehensive biological knowledge integration, and the prediction models mostly rely only on cell line gene expression data. Given the abundance of data in recent years, more and more models have begun to combine cell line multi-omics with drug chemical structure characteristics.^20^

In preclinical DRP research, building a comprehensive framework to improve prediction accuracy is a key goal, focusing on effectively extracting drug and cell line features and their combination for prediction. A variety of methods have been used for drug feature extraction to improve drug characterization, such as DeepDRA^21^ using deep neural networks (DNNs) to extract molecular descriptors and fingerprints, tCNNS^22^ using convolutional neural networks (CNNs) to encode SMILES string sequences, DeepCDR^23^ and GraphDRP^24^ using graph neural networks (GNNs) to learn molecular structure information, etc. For cell line feature extraction, due to the complexity and high dimensionality of omics data, effective extraction faces challenges. DNN, CNN, and autoencoder (AE) are widely used to extract features from single-omic or multi-omics data, such as SWnet^25^ using one-dimensional (1D) CNNs to extract expression and mutation features, DeepDSC^26^ applying stacked attention mechanism (AE) to encode gene expression features, etc. From the perspective of drug sensitivity mechanism, CDR prediction models also consider features from pathway information, drug-target data, and protein-protein interactions (PPIs). For example, HiDRA^27^ introduces a hierarchical attention network model to represent biological pathways and their member genes related to drug response. PathDNN^28^ reconstructs the classic DNN architecture by introducing a pathway node layer connected to the input gene and drug-target nodes. However, PathDNN only uses the confidence score from the Search Tool for Interactive Chemistry (STITCH) as the drug-target interaction (DTI) to represent drug features and can only be used for molecules in the STITCH database.^29^ In addition, single-cell data are also a potential source of cell line features. For example, the CellLM^30^ model based on single-cell RNA sequencing (scRNA-seq) data shows enhanced semantic representation of cell line data, but the study mainly focuses on single-cell tasks and does not fully explore drug response research in cell lines.

The field of graph-based learning has received widespread attention and has promoted the rapid development of its application in the field of bioinformatics. These technologies have shown significant practicality in understanding complex biological systems, but in DRP, most methods are mainly carried out within the scope of undirected bipartite networks. For example, GraphSAGE^31^ learns a node representation method by sampling local neighbors of a vertex and aggregating vertex features, but it can only use uni-modal features. Graphcdr^32^ constructed a graph neural network with contrastive learning to enhance the generalization ability of DRP, and TGSA^33^ proposed a framework consisting of a twin graph neural network and a similarity enhancement algorithm to predict drug response. Generally, a comprehensive study of drug response requires separate analysis of drug sensitivity and resistance, so it is necessary to improve the directed bipartite network algorithm. HGCL-DR^34^ proposes a heterogeneous graph contrastive learning framework that captures local-global associative features via a bidirectional graph convolutional network (GCN), graph diffusion, and contrastive learning. MMGIN^35^ is designed with a multi-modal graph isomorphism network (GIN) for multi-task learning. Based on compound fingerprints and molecular graphs, the model learns the corresponding representations through a two-channel structure. MMDDI-SSE^36^ integrates drug sequence modality with DDI graph representations. This architecture uses static subgraph generation to capture structural properties, adopts a graph AE to learn local and global topological features from the subgraphs, and meanwhile processes various sequence-based characteristics.

The rich omics data of cancer cell lines have increased the complexity of research, and in-depth exploration of their impact on predictive models has become indispensable. At present, researchers have made great progress in using graph structure information to predict cancer drug sensitivity. In the relevant graph structure, each node corresponds to a specific cell line or drug and is unique in the feature space.^37^ However, most existing methods treat the cell line-drug bipartite network as an undirected graph, but in fact, the edges in the network reflect sensitivity or resistance, and in essence, it should be a directed graph. In view of this, it is crucial to incorporate heterogeneous and directed characteristics. From a graph-theoretic perspective, neglecting directionality can obscure distinct community structures, as demonstrated in statistical network analysis.^38^ Translating this topological insight to the biological context, the asymmetry in interactions between different nodes contains key structural information.

**Figure 1:**
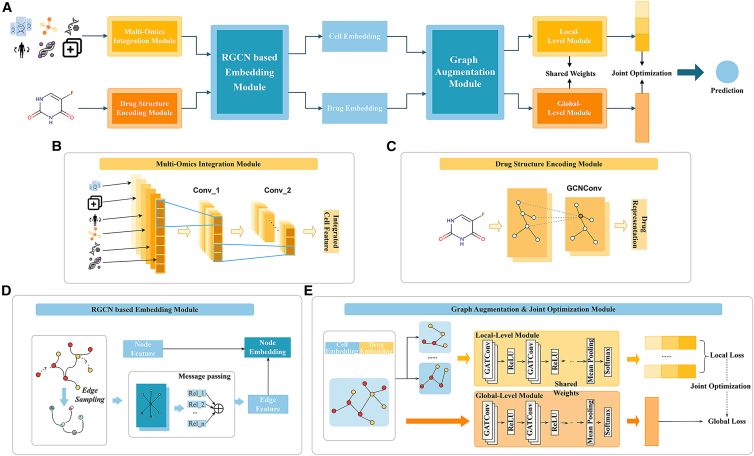
The overview of MOGA (A) The architecture of MOGA consists of four main components: the multi-omics integration module, the drug structure encoding module, the RGCN-based embedding module, and the graph augmentation and joint optimization module. (B) The multi-omics integration module employs a convolution network to integrate multi-omics data of cell lines. (C) Steps for generating drug substructure encoding using graph convolution network. (D) Using RGCN to encode the heterogeneous graph and obtain the embedding matrix of the node. (E) Graph augmentation and join optimization module. Using local-level GAT to encode the augmented graph, and using global-level GAT to encode the original graph, and then jointly optimize the two levels.

This article introduces a DRP method called MOGA (Figure 1). MOGA integrates multiple types of genomic data, including transcriptomics, proteomics, copy number variations, mutations, DNA methylation, and metabolomics data, to capture the complex biological information in drug responses. Additionally, MOGA uses relational graph convolutional networks (RGCNs) to capture relational information in the drug response graph. It then employs graph augmentation with sensitive and non-sensitive nodes and, through a joint optimization strategy, eliminates the impact of non-sensitive nodes. Our main contributions include the following.

(1) Multi-omics integration with modality-specific convolution: unlike most existing models that rely on simple concatenation or uniform weighting of multi-omics data, MOGA adopts a dedicated convolutional network for each omics type. This design captures modality-specific biological features before fusion, avoiding information loss and maximizing the synergy between different omics layers.
(2) Type-specific graph augmentation strategy: we propose a graph augmentation technique that categorizes neighboring nodes into sensitive and non-sensitive types. By applying targeted perturbation only to non-sensitive nodes, MOGA preserves critical biological interactions while reducing noise interference; this is distinct from uniform perturbation methods that may destroy useful structural information.
(3) Directed heterogeneous graph modeling with RGCN: MOGA models the drug-cell line interaction network as a directed graph and employs RGCN to capture relationship-specific features. This addresses the limitation of existing methods that treat the network as undirected, aligning with the directional nature of biological responses.
(4) Joint optimization for stability and accuracy: the integration of graph augmentation with a dual-loss objective ensures the model’s robustness to non-sensitive information changes while maintaining high prediction accuracy, an approach not explored in previous graph-based DRP models.

## Results

### Datasets

This study focuses on the field of cancer and integrates two core databases: the GDSC and the CCLE. The CCLE provides multi-omics data, including genomics, transcriptomics, proteomics, and epigenomics, but has limited drug response data. In contrast, the GDSC focuses on drug responses and covers information on drug sensitivity and resistance in various cancer cell lines. As shown in Table 1, initially, we compiled data on 1,406 cancer cell lines and 449 drugs, classifying drug responses into three categories: sensitivity, resistance, and no correlation. Following a rigorous cleaning and alignment process, we focused on a core subset of 192 cell lines characterized by complete multi-omics data, including transcriptomics, proteomics, copy number variations, mutations, DNA methylation, and metabolomics. For these samples, drug response labels were sourced from GDSC, while drug molecular structures (SMILES) were retrieved from PubChem. After rigorous preprocessing and data alignment, the finally organized dataset contains 192 cancer cell lines and 352 types of drugs. To classify drug responses, we set two thresholds based on IC50 values (log10-transformed): cases with an IC50 value <−3 were categorized as sensitive responses, while those with an IC50 value >3 were regarded as resistant responses. In the subsequent stage of our study, we compiled a large-scale dataset consisting of 29,697 drug response entries, among which 3,001 were classified as sensitive responses and 26,696 as resistant responses. Specifically, we investigated the complex drug response relationships within a directed graph framework, which was represented using two adjacency matrices. One adjacency matrix represents sensitivity, where a value of 1 indicates a sensitive response, while the other matrix represents resistance, where a value of 1 indicates a resistant response. Based on this, 10% of all drug-cell line combinations were constructed as the test set to ensure the comprehensiveness and reliability of the study, where neither the drugs nor the cancer cell lines in the test set overlap with those in the training set or the validation set.

**Table 1:** A statistical summary of our datasets

Type	Specification	Number of samples
Drug	–	352
Cell line	–	192
Cell line-omics (total of six types of relationships)	transcriptomics	16,382
	proteomics	12,755
	copy number variation	16,382
	mutations	16,381
	DNA methylation	14,234
	metabolomics	225
Response (drug-cell line)	sensitive	3,001
	resistant	26,696

### Experiment setting

During our experiments, we focused on exploring the following key questions:

RQ1: performance comparison: how does MOGA perform compared to the state-of-the-art prediction methods in the task?

RQ2: impact of multi-omics data: what changes does the integration of multi-omics data, such as transcriptomics and metabolomics, bring to the prediction results? What impact do different omics data have on the prediction outcomes?

RQ3: analysis of graph augmentation strategy: what impact does the graph augmentation strategy have on the results, and how do different perturbation levels during sampling affect the performance of the augmentation strategy?

In our prediction experiments, we conducted performance comparisons between MOGA and the most advanced current methods to measure the reliability and efficiency of MOGA under different circumstances. Meanwhile, to ensure the reliability of the research results, we used 5-fold cross-validation for rigorous testing.

### Baseline models

By benchmarking against the following well-validated state-of-the-art methods GraphDRP, MOFGCN, SRMF, TGSA, Heterogeneous Graph Transformer (HGT), REDDA, and DRExplainer, we conducted a comprehensive evaluation of the model’s performance. All baseline models adopt the optimal parameter settings in the original paper. The details are as follows:

GraphDRP^24^ utilizes various graph neural network models to process the SMILES sequences of drugs, while employing convolution neural networks to analyze the molecular feature profiles of cancer cell lines.

GraphSAGE^31^ extends GCN to inductive learning tasks by training functions for aggregating node neighbors (i.e., convolutional layers), enabling generalization to unseen nodes.

MOFGCN^20^ utilizes a multi-scale network integration strategy to integrate the chemical characteristics of drugs with the genetic features of cell lines. It provides a comprehensive analysis of the complex interactions between drugs and cell lines, constructing a precise analytical model based on multi-dimensional information integration to achieve an accurate depiction of drug mechanisms of action.

SRMF^39^ integrates the similarity information of drugs and cell lines to improve prediction accuracy and, more efficiently, infers the response of cell lines to specific drugs by leveraging the relationships between similar drugs and cell lines.

TGSA^33^ leverages the complexity of PPI networks to accurately construct high-quality representations of cell lines, which is crucial for precise DRP based on gene expression profiles.

HGT^40^ designs parameters related to node and edge types for each edge to characterize its heterogeneous attention. This method is capable of maintaining exclusive representation methods for different types of nodes and edges.

REDDA^41^ is a method that incorporates three types of attention mechanisms. It sequentially learns the representations of different nodes through a node-embedding block based on a general heterogeneous graph convolution network, a topological subgraph embedding block, a graph attention block, and a layer attention block.

DRExplainer^42^ constructs a directed bipartite network that integrates the multi-omics features of cell lines, the chemical structures of drugs, and known drug responses to achieve directed prediction. On this basis, it further learns the subgraphs in the directed bipartite network that are most relevant to each prediction.

HGCL-DR^34^ is a heterogeneous graph contrastive learning framework that effectively integrates both global and local feature representations for drug repositioning.

MMGIN^35^ incorporates a multimodal representation learning model to acquire a comprehensive compound representation. This model adopts a two-channel structure to independently learn fingerprint representation and molecular graph representation.

MMDDI-SSE^36^ integrates drug sequence modality with DDI graph representations that employ static subgraph generation to capture structural properties.

### Prediction model comparison (RQ1)

Table 2 shows the model’s performance across various metrics, highlighting the superior capabilities of our model. AUC is an effective way to measure the overall performance of a model, as it provides a comprehensive reflection of the model’s strengths and weaknesses. Its unique advantage lies in its complete independence from specific classification thresholds. On the other hand, AUPR focuses more on the delicate balance between precision and recall at different thresholds, offering a more nuanced and in-depth evaluation. For dealing with imbalanced datasets, AUPR holds an irreplaceable value. Both metrics play a crucial role in the comprehensive assessment of classification models. The higher their values, the stronger the model’s ability in class distinction and prediction accuracy.

**Table 2:** The performance of MOGA and baseline models

Model	AUC	AUPR	Precision	Recall	F1 score
GraphDRP	0.8862 ± 0.013	0.8425 ± 0.014	0.7852 ± 0.016	0.8724 ± 0.014	0.8202 ± 0.015
GraphSAGE	0.823 ± 0.019	0.786 ± 0.020	0.7731 ± 0.011	0.8081 ± 0.016	0.7971 ± 0.025
MOFGCN	0.8404 ± 0.015	0.6440 ± 0.018	0.7233 ± 0.017	0.8312 ± 0.016	0.7951 ± 0.018
SRMF	0.7905 ± 0.017	0.7448 ± 0.016	0.6928 ± 0.018	0.7897 ± 0.018	0.7048 ± 0.017
TGSA	0.9108 ± 0.011	0.9203 ± 0.009	0.8855 ± 0.013	0.8980 ± 0.012	0.9021 ± 0.024
HGT	0.9388 ± 0.008	0.8392 ± 0.012	0.6528 ± 0.014	0.9155 ± 0.011	0.9086 ± 0.016
REDDA	0.9001 ± 0.011	0.4280 ± 0.021	0.4657 ± 0.017	0.4329 ± 0.018	0.4480 ± 0.015
DRExplainer	0.8900 ± 0.012	0.8592 ± 0.013	0.7720 ± 0.014	0.8651 ± 0.013	0.7841 ± 0.009
HGCL-DR	0.9472 ± 0.007	0.8945 ± 0.009	0.8611 ± 0.013	0.9151 ± 0.010	0.8622 ± 0.010
MMGIN	0.9172 ± 0.010	0.8255 ± 0.013	0.8731 ± 0.010	0.8395 ± 0.010	0.8562 ± 0.013
MMDDI-SSE	0.9378 ± 0.008	0.8921 ± 0.009	0.8943 ± 0.013	0.9087 ± 0.011	0.9126 ± 0.011
MOGA	0.9579 ± 0.006	0.9013 ± 0.008	0.9114 ± 0.012	0.9504 ± 0.009	0.9257 ± 0.009

Our model demonstrates superior performance on both AUC and AUPR metrics, significantly outperforming other state-of-the-art (SOTA) models. Specifically, its AUC and AUPR score is higher than that of the highest performing model HGCL-DR. These results fully demonstrate the excellent performance and strong robustness of our model in the classification task.

In addition, our model performs well in terms of specificity, which is particularly crucial for analyses dominated by negative samples. Meanwhile, compared with other models, our model also achieves a good F1 score. It is worth noting that, as mentioned earlier, our model excels in dealing with complex biological problems. MOGA can not only routinely predict drug sensitivity but also accurately identify drug resistance and non-significant associations, highlighting its significant contribution to the field of drug response research.

**Figure 2:**
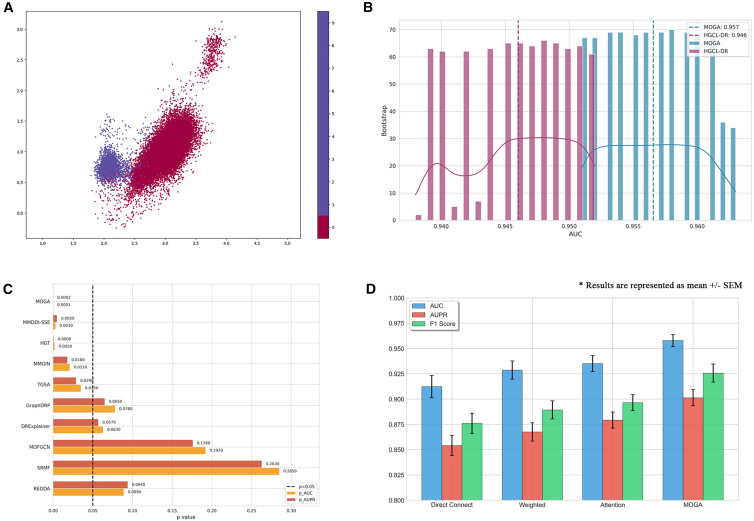
The performance of MOGA (A) Visualization of embedding for sensitive and non-sensitive samples. (B) Delong test for AUC between MOGA and HGCL-DR. (C) *p* values of AUC and AUPR using bootstrap. (D) The results of diffient multi-omics integration strategies.

Furthermore, to provide an intuitive visualization of the spatial distribution of positive and negative node embedding in the drug response network after integrating multi-omics information, we employed the uniform manifold approximation and projection (UMAP) dimensional reduction technique. This method clearly reveals the distribution characteristics of node embedding in the space. Figure 2A distinctly displays the two classes of nodes, thereby confirming the effectiveness of our proposed approach.

We added cross-dataset validation using the CTRP dataset, and we have also conducted verification experiments based on TCGA clinical patient data. Specifically, for TCGA, we mapped the patients’ omics data to the omics data of CCLE cell lines by performing feature normalization and further tested the clinical response prediction performance. During the testing, only drugs that have not appeared in GDSC were used. We fine-tuned MOGA on CCLE+GDSC and tested on CTRP. As shown in Table 3, compared with the two top-performing baseline models, MOGA has achieved excellent performance on two independent test sets, which confirms its strong generalization ability. It also exhibits promising performance in predicting responses to new drugs, which is of great significance for the clinical translation of the model.

**Table 3:** Results of cross-dataset validation

Model	CTRP			TCGA		
	AUC	AUPR	F1 score	AUC	AUPR	F1 score
HGCL-DR	0.885 ± 0.021	0.813 ± 0.025	0.832 ± 0.024	0.832 ± 0.024	0.785 ± 0.026	0.801 ± 0.023
MMDDI-SSE	0.879 ± 0.023	0.801 ± 0.027	0.825 ± 0.026	0.827 ± 0.025	0.778 ± 0.027	0.795 ± 0.024
MOGA	0.912 ± 0.018	0.857 ± 0.023	0.876 ± 0.021	0.896 ± 0.021	0.832 ± 0.023	0.854 ± 0.020

We also performed the DeLong test for AUC and calculated the *p* values of AUPR based on bootstrap sampling. All experiments were conducted with 3 random seeds, with the results presented in Figures 2B and 2C. As shown in Figure 2B, the AUC value of MOGA is higher than that of the optimal baseline (HGCL-DR), and it can also be seen from the trend of the curve that the AUC distribution of MOGA is more stable. Figure 2C presents the comparison of *p* values for AUC and AUPR between the optimal baseline (HGCL-DR) and other models, from which it can be concluded that the predictive performance of MOGA is reliable.

### Multi-omics data study (RQ2)

We plan to address the following two key questions in our work.

(1) Is using multi-omics data more effective than using single-omics data?
(2) Is a specific type of omics data more suitable for drug synergy prediction?

To this end, we first designed comparative experiments to compare the omics type-based convolution integration method proposed in this paper with three mainstream multi-omics integration methods. We retrained MOGA using each integration method and evaluated the performance through 5-fold cross-validation on the test set. As shown in Figure 2D, the integration method of MOGA outperforms existing methods. This is because omics type-based convolution can effectively capture modality-specific features of each omics before integration, avoiding information loss caused by direct concatenation or inappropriate weighting.

**Figure 3:**
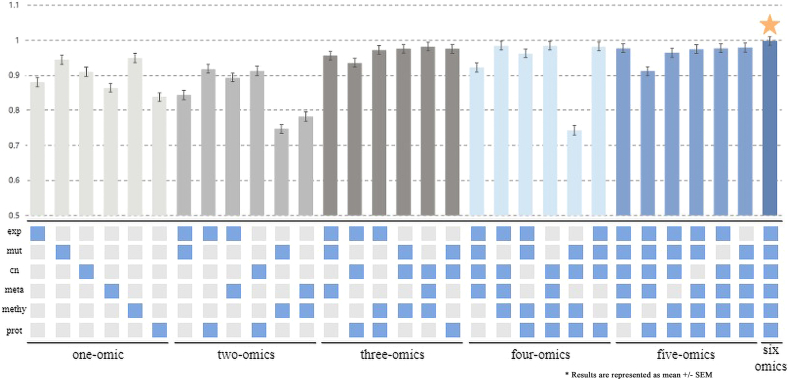
AUC scores for experiments with different combinations of omics data Blue squares indicate the types of omics data used.

Then, we have conducted an in-depth investigation into the impact of different combinations of omics data. MOGA initially incorporated six types of omics data. We randomly selected six combination schemes, covering various types of omics data. Figure 3 intuitively displays the different omics combinations and their corresponding experimental results.

In the single-omics and dual-omics experiments, the model performance is significantly affected by the type of omics data. No matter how they are combined, the results will not be very good. It is worth noting that when using transcriptomics, copy number variation, or proteomics data, the MOGA model will perform relatively better, which highlights the superiority of the MOGA architecture.

As the number of multi-omics data types increases, the model’s predictive ability is significantly enhanced. When all six types of omics data are used, the model achieves the best predictive performance. Moreover, MOGA is not particularly sensitive to specific types of omics data. As shown in Figure 3, when the number of omics types exceeds two, the predictive results plateau. This finding is consistent with a recent large-scale benchmark study,^43^ which demonstrated that integrating extensive multi-omics modalities does not strictly guarantee performance gains. This is likely because different omics layers (e.g., mRNA and miRNA) are biologically interdependent and share significant information, meaning that two or three complementary modalities are often sufficient to capture the underlying patterns.

### Augmentation graph analysis (RQ3)

The aforementioned experiments indicate that MOGA exhibits good and reliable performance, and the graph augmentation strategy plays a crucial role. To further verify the impact of this graph augmentation strategy, we designed comparative experiments to explore its effectiveness.

**Figure 4:**
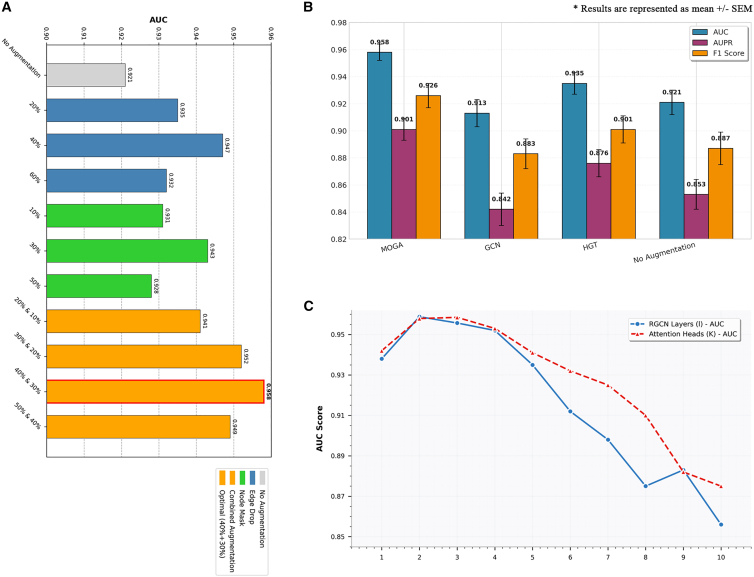
Performance of ablation experiment (A) AUC results of different graph augmentation strategies. (B) Comparative experimental results of different graph embedding models. (C) Sensitivity analysis results of RGCN layer number and attention head number.

To verify the core value of the graph augmentation strategy in MOGA; clarify the impacts of different augmentation types, including edge deletion, feature masking, and combined augmentation, and augmentation rates on model performance; and screen out the optimal augmentation scheme, we designed comparative experiments with different augmentation types and augmentation rates. As shown in Figure 4A, edge deletion reduces the interference of noise on the model by randomly removing some non-critical connections, such as false drug-cell line non-responsive edges and low-similarity drug/cell line connections. The model achieves the optimal performance when the augmentation rate is 40%—at this rate, redundant edges are eliminated while core biological relationships (sensitive/resistant edges and high-similarity edges) are preserved. When the augmentation rate is lower than 20%, noise removal is insufficient, leading to limited performance improvement; when it exceeds 60%, excessive deletion results in the loss of core edges (e.g., some real sensitive edges are mistakenly deleted), making the model unable to learn the complete drug-cell line interaction network and thus causing performance degradation.

Feature masking simulates missing or noisy scenarios in biological data by randomly masking some feature dimensions of nodes (such as a certain type of omics feature of cell lines and part of the molecular fingerprint of drugs), forcing the model to learn more robust core features. A masking rate of 30% yields the best performance: at this rate, the model will not be insufficiently constrained due to an excessively low masking rate (<10%), nor will it lose key features (e.g., the core functional group features of drugs and the key mutation features of cell lines) due to an excessively high masking rate (>50%).

The combined scheme of edge deletion (40%) + feature masking (30%) achieves the optimal performance, with the AUROC increased by 4.02% compared with the scenario without augmentation, and it significantly outperforms single augmentation types. This result is consistent with the characteristics of biological data; the drug-cell line interaction network has connection redundancy addressed by edge deletion, while multi-omics and molecular features have noise interference addressed by feature masking. The synergistic effect of the two can optimize the quality of input data more comprehensively, allowing the model to focus on sensitive nodes and core features.

To verify the core value of the RGCN in MOGA, we replaced RGCN with GCNs (which do not distinguish between relationship types) and HGTs, aiming to clarify RGCN’s advantage in adapting to directed heterogeneous graphs.

As shown in Figure 4B, the core limitation of GCN lies in its undirected and homogeneous modeling capability. However, the directed heterogeneous graph in MOGA contains three types of edges with distinct functions. GCN treats all edges uniformly as undirected edges; it can neither distinguish the biological opposition between “sensitive” and “resistant” edges nor utilize the directional information of edges. This prevents the model from learning accurate drug-cell line response relationships, resulting in the most significant performance loss among the three modules.

Although HGT supports heterogeneous graph modeling, it dynamically learns the importance of nodes or edges through an attention mechanism rather than explicitly modeling relationship types. In the DRP task, the biological significance of sensitive and resistant edges is clear and fixed. By assigning independent weights to different relationship types, RGCN can more directly capture such fixed functional differences. In contrast, HGT’s attention mechanism may overly focus on nodes with high connectivity (e.g., high-frequency drugs/cell lines) and ignore low-connectivity yet critical sensitive/resistant edges, leading to performance degradation.

Compared with AUC, AUPR is more sensitive to imbalanced data. This further proves that relationship type modeling can help the model better identify minority-class sensitive samples and avoid minority-class prediction bias caused by edge-type confusion.

Figure 4C presents the results of the parameter sensitivity analysis. From the perspective of the number of RGCN layers (l), the model achieves optimal performance when l = 2. When l < 2, information propagation is insufficient, making it impossible to capture complex relationships between nodes; when l > 2, the over-smoothing phenomenon reduces the distinguishability of node embeddings. MOGA demonstrates robustness when l∈[2,3].

In terms of the number of attention heads (K), the model balances information diversity and computational efficiency when K = 3. After K exceeds 3, there is no significant improvement in performance, but the computational cost increases, which confirms that the model exhibits robustness when K∈[2,4].

We also analyzed the computational efficiency of MOGA and proposed potential optimization suggestions for large-scale datasets. Details can be referred to in Table S2 and Data S1.

### Case study

We trained MOGA using the known cell line-drug response data in the training set and then evaluated its predictive performance on the test set, ranking the predictions by their scores. Table 2 lists the top-scoring predicted sensitivity cell lines by MOGA.

As shown in Table 4, most of the results can be validated in the existing literature on PubMed. For example, the drug GSK690693 has been confirmed to be sensitive to the cell line RCHACV.^44^ In addition, dasatinib has also been confirmed by multiple studies to be sensitive to a variety of cell lines, including RCHACV,^45^ CAL51,^46^ and SUDHL6.^47^ Taking the DLBCL cell line SUDHL6 as an example, studies have shown that dasatinib can inhibit multiple SRC kinases simultaneously, demonstrating good therapeutic effects on lymphoma cells *in vitro*, and this sensitivity is not limited by molecular subtypes.^47^

**Table 4:** Top-scoring predicted sensitive cell lines of three drugs

Drug	Cell line	Evidence
Bortezomib	SW837	PMID: 23011889^48^
	OE33	N/A
	UACC257	PMID: 31019203^49^
	HCC1143	N/A
	CCK81	N/A
	MFE296	PMID: 39808525^50^
	JHH4	N/A
GSK690693	RCHACV	PMID: 19064730^44^
	CMK	N/A
	EFM192A	N/A
	NCIH23	N/A
Dasatinib	RCHACV	PMID: 23153538^45^
	SUDHL4	N/A
	CAL51	PMID: 17268817^46^
	SUDHL6	PMID: 29567799^47^

**Figure 5:**
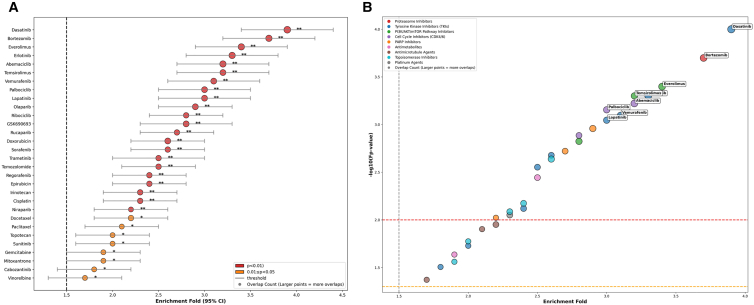
Results of enrichment analysis (A) Enrichment characteristics of high-attention neighbors of drugs with known drug targets/pathways. (B) Bubble chart analysis of drug high-attention neighbor enrichment.

As shown in Figure 5A, among the 30 drugs, 28 (93.3%) had an enrichment fold ≥1.5, of which 22 showed significant enrichment (*p* < 0.01). No drug showed enrichment, confirming that the high-attention neighbors of MOGA are highly correlated with the known mechanisms of action of drugs. For core drugs (e.g., dasatinib, bortezomib, and everolimus), the enrichment fold was ≥3.0 with narrow confidence intervals, indicating that the model has strong stability in target prediction for such drugs. The enrichment folds of chemotherapeutic drugs such as paclitaxel and gemcitabine ranged from 1.7 to 2.1, which were lower than those of targeted drugs. This is consistent with the biological rule that targeted drugs have more definite mechanisms of action and more concentrated targets.

As shown in Figure 5B, core drugs (such as dasatinib, bortezomib, and everolimus) are concentrated in the upper-right region, with large bubbles (overlap count ≥8), confirming that the high-attention neighbors of these drugs are highly consistent with known targets/pathways. Tyrosine kinase inhibitors (TKIs) and PI3K/AKT/mTOR pathway inhibitors generally exhibit better enrichment performance than traditional chemotherapeutic drugs (e.g., antimetabolites and platinum-based drugs), which is consistent with the mechanism-of-action specificity of targeted drugs. No drugs fall into the lower-left region, further verifying the biological relevance of the high-attention neighbors identified by the MOGA model.

## Discussion

Nowadays, heterogeneous graphs that incorporate both drugs and cell lines have become powerful tools for predicting drug responses; however, graph neural network models based on multi-omics data have yet to fully unleash their vast potential. In the field of precision medicine, stakeholders require not only accurate predictions but also a comprehensive understanding of the model’s decision-making process. To meet this demand, our study proposes MOGA, a joint optimization strategy that integrates multi-omics data with graph augmentation techniques. By incorporating multi-omics information, the model significantly improves predictive performance while accounting for potential sensitive relationships between nodes in the graph. This approach reduces interference from non-sensitive information during perturbation and preserves critical sensitive node details. It is the synergistic effect of these key factors that enables our model to demonstrate exceptional performance across multiple datasets.

To validate the stability of the proposed model, we conducted comparative experiments between MOGA and current SOTA methods. The results demonstrated that MOGA achieved excellent performance on both AUROC and AUPR evaluation metrics. Furthermore, through ablation studies, we further confirmed the core role of MOGA in DRP tasks. Additionally, we employed case studies to verify the effectiveness of multi-omics data in identifying potential DRP and conducted in-depth investigations into the impact of different omics data and their combinations on the results, thereby providing a more comprehensive understanding of the unique advantages of multi-omics data in DRP tasks.

In the future, we plan to integrate self-supervised learning, for instance, contrastive learning for missing value imputation to enhance MOGA’s tolerance to incomplete data, and we will extend MOGA into a dynamic GCN, which will incorporate IC50 values across multiple doses and temporal multi-omics data, such as changes in gene expression after drug treatment, while fusing patients’ clinical data and real-world evidence to improve its clinical applicability. We also integrate attention visualization and pathway enrichment analysis to identify key biological mechanisms driving drug sensitivity, thereby enhancing clinical trust in the model.

### Limitations of the study

Despite the significant success of the MOGA model, there are still some limitations that need to be addressed. When multi-omics data are incomplete or contains noise, the performance of MOGA decreases significantly. Furthermore, MOGA models drug responses as static binary outcomes (sensitive/resistant) and fails to capture dynamic changes, such as dose-dependent responses or acquired resistance over time. In addition, current validation relies on cell line data (CCLE/GDSC/CTRP), and translation to clinical applications in patients requires the integration of clinical data and prospective clinical trials.

## Resource availability

### Lead contact

Requests for further information and resources should be directed to and will be fulfilled by the lead contact, Tianyi Zang (tianyi.zang@hit.edu.cn).

### Materials availability

This study did not generate new unique reagents.

### Data and code availability

• The data reported in this paper are publicly available on GitHub (https://github.com/kyzhang22/MOGA).
• All original code has been deposited at GitHub and is publicly available as of the date of publication. The DOI is listed in the key resources table.
• Any additional information required to reanalyze the data reported in this paper is available from the lead contact upon request.

## Acknowledgments

This work was supported by the Flexible Introduction of Leading Talents under the 2023 Kunlun Talents High-End Innovation and Entrepreneurship Talents Project of Qinghai Province (QHKLYC-GDCXCY-2023-320), Qinghai University Research Ability Enhancement Project (2025KTSA01), “Unveiling the Leader” Science and Technology R&D Projects (2022ZXJ03C06), 10.13039/501100001809National Natural Science Foundation of China (62076082), and National Key Research and Development Project (2016YFC0901605).

## Author contributions

K.Z. designed the study, wrote the manuscript, and analyzed the results. R.W. curated the data and helped revise the manuscript. T.Z. and Y.Z. participated in method design and manuscript writing.

## Declaration of interests

The authors declare no competing interests.

## STAR★Methods

### Key resources table

**Table:** 

REAGENT or RESOURCE	SOURCE	IDENTIFIER
**Deposited data**		

Drug response data	GDSC^5^	https://www.cancerrxgene.org/
Drug response data	CTRP^7^	https://portals.broadinstitute.org/ctrp.v2/
Multi-Omics data	CCLE^6^	https://sites.broadinstitute.org/ccle/
Cancer genomic data	TCGA^8^	https://www.cancer.gov/ccg/research/genome-sequencing/tcga

**Software and algorithms**		

GraphDRP	Nguyen et al.^24^	https://github.com/hauldhut/GraphDRP
GraphSAGE	Hamilton et al.^31^	https://github.com/williamleif/GraphSAGE
MOFGCN	Wei et al.^20^	https://github.com/weiba/MOFGCN
SRMF	Wang et al.^39^	https://github.com/linwang1982/SRMF
TGSA	Zhu et al.^33^	https://github.com/violet-sto/TGSA
HGT	Hu et al.^40^	https://github.com/acbull/pyHGT
REDDA	Gu et al.^41^	https://github.com/gu-yaowen/REDDA
DRExplainer	Shi et al.^42^	https://github.com/vshy-dream/DRExplainer
HGCL-DR	Wang et al.^34^	https://github.com/Tomchen1231/HGCL-DR
MMGIN	Wang et al.^35^	https://github.com/528711/MMGIN
MMDDI-SSE	Wang et al.^36^	https://github.com/Tomchen1231/MMDDI-SSE
MOGA	This paper	https://github.com/kyzhang22/MOGA

### Method details

#### Multi-omics data integration module

Inspired by previous research, we developed a specific convolution neural network to integrate multi-omics data (Figure 1B), aiming to provide a unique representation for each cancer cell line. These data comprehensively describe the multi-omics features of cancer cell lines, including transcriptomics, proteomics, copy number variations, mutations, DNA methylation, and metabolomics feature vectors, denoted as c_t_,c_p_,c_c_,c_m_,c_d_,c_e_. Subsequently, these vectors are transformed into an F-dimensional vector embedding through the Equation 1:

(Equation 1) $$\begin{array}{c} c=\text{concat}\left[g_{t}\left(c_{t}\right),g_{p}\left(c_{p}\right),g_{c}\left(c_{c}\right),g_{m}\left(c_{m}\right),g_{d}\left(c_{d}\right),g_{e}\left(c_{e}\right)\right] \end{array}$$

where c∈R^F^ is used to denote the feature vector of cancer cell lines. The terms {g_t_,g_p_,g_c_,g_m_,g_d_,g_e_} represent different omics feature transformation convolution network layers. For a set of cancer cell lines $C={\left\{c_{i}\right\}}_{i=1}^{n_{c}}$, n_c_indicates the number of cell lines, and the features of all cancer cell lines are ultimately integrated into a feature matrix $C\in R^{n_{c}\times F}$, where F denotes the dimension of the multi-omics feature matrix.

Distinctive Design Compared to Existing Methods: Most existing models rely on single-omics data or simple concatenation of multi-omics features, which fail to capture synergistic biological information across omics layers. In contrast, MOGA adopts a dedicated convolution network for each omics type to learn type-specific feature representations, then fuses them via concatenation. The structural information of the Graph Convolutional Network (GCN) refers to Supplementary Materials Table S1.

#### Drug structure encoding module

The study takes the SMILES format of a drug as input and converts it into a molecular graph structure, where nodes represent atoms and edges represent chemical bonds between atoms. (Figure 1C) The molecular graph of a specific drug is denoted as G_d_=(X_d_,A_d_), where $X_{d}\in R^{n_{d}\times F}$ is the node attribute matrix containing atomic feature information and $A_{d}\in R^{N_{d}\times N_{d}}$ is the adjacency matrix recording the types of chemical bonds between atoms and their connection. N_d_ represents the number of atoms in the molecular graph of drug d. In this study, a Graph Neural Network (GNN) encoder φ(·) is used to capture the latent feature representations of atomic nodes. For a given drug sets $D={\left\{d_{i}\right\}}_{i=1}^{N_{D}}$, where N_D_ is the total number of drugs, the GNN encoder is applied to extract features from all drugs to obtain the comprehensive representation $D\in R^{N_{D}\times F}$.

MOGA directly models drugs as molecular graphs and uses GCN to learn hierarchical structural features. This design preserves the 2D topological information of drugs, ensuring more accurate chemical feature representation.

#### RGCN-based embedding module

As shown in Figure 1D, given the need to capture complex structural information in the network, we adopted the Relational Graph Convolution Network(RGCN). RGCN is a powerful framework specifically designed for modeling graph data containing multiple types of relationships. With its superior performance, RGCN effectively facilitates the propagation of similarity relationships and reaction information, thereby revealing the complex and diverse relationships between cell lines and drugs.

Under this framework, we propagate and aggregate information through directed edges to follow the natural directionality of biological and chemical entities. Our model iteratively updates node representations in the directed multi-graph G=(V,E,R), which contains a vertex set V, an edge set E, and a relation set R. As mentioned earlier, the directed heterogeneous network not only covers the sensitive interactions between cell lines and drugs but also includes the non-sensitive interactions between drugs and cell lines. Therefore, this directed multi-graph is composed of two different types of edges. The previously learned cell line representations C and drug representations D are combined to form the node attribute matrix $X=\left[\begin{array}{c} C\\ D \end{array}\right]\in R^{\left(N_{c}+N_{d}\right)\times F}$ in the graph.

The RGCN model is specifically designed to handle the complexity of data with multiple relational features, making it highly suitable for prediction tasks in heterogeneous graphs involving various relationships between drugs and cell lines.^51^ As shown as Equation 2 and Equation 3, the propagation model updates the forward pass information of each entity v_i_ iteratively, taking into account the relations and their corresponding links:

(Equation 2) $$\begin{array}{c} h_{i}^{l+1}=\sigma \left(\sum_{r\in R}\;\sum_{j\in N_{i}^{r}}\frac{1}{c_{i,r}}W_{r}^{l}h_{j}^{l}+W_{0}^{l}h_{i}^{l}\right) \end{array}$$

(Equation 3) $$\begin{array}{c} H_{i}=\frac{1}{p}\sum_{i\in p}h_{i}^{l} \end{array}$$

where R is used to describe the different connection types between nodes. $N_{i}^{r}$ denotes the set of neighboring nodes of the given node v_i_ for a specific relation r. C_i,r_ is a constant. Similar to the Graph Convolution Network framework, h^(l)^ represents the hidden layer of node v in the l-th layer of the neural network. The weight matrix W is used to transform input features or hidden layer outputs. σ(·) is an activation function applied element-wise, used to introduce non-linearity into the model. $H\in R^{n\times d}$ is the embedding matrix and p represents different sources of features.

Most baseline methods model cell line-drug interactions as undirected bipartite graphs, which ignore the directional nature of drug responses (e.g., “cell line A is sensitive to drug B” is not equivalent to “drug B is sensitive to cell line A”). RGCN can distinguish between sensitive and resistant edges via relation-specific weight matrices. This design aligns with biological reality, as sensitivity and resistance are mutually exclusive and direction-dependent phenotypes.

#### Graph augmentation and joint optimization module

To enhance the model’s ability to capture sensitive variables, we propose a new augmentation strategy. (Figure 1E) In the original drug response graph, the neighbors of a node are divided into two categories: the sensitive node set S_s_, which has a significant impact on the classification results and the non-sensitive node set S_n_, which has a relatively smaller impact on the classification results, with no interference between the two types of nodes. Specifically, before graph embedding encoding, we randomly mask specific dimensions of some node embedding or some connections between nodes in the original graph, thereby introducing random noise to the forward graph before each training session.

During the graph augmentation process, we expand the nodes and features of the augmented graph. Taking two augmented graphs as examples, in G_1_, the sensitive node set is labeled as S_s1_, and the non-sensitive node set is labeled as S_n1_. In G_2_, the sensitive node set is labeled as S_s2_, and the non-sensitive node set is labeled as S_n2_. Equation 4 is the definition of the augmented graph:

(Equation 4) $$\begin{array}{c} G_{1}=\left(A_{1},H_{1}\right)\;G_{2}=\left(A_{2},H_{2}\right) \end{array}$$

where $A\in R^{N_{G}\times N_{G}}$ is the adjacency matrix, $H\in R^{n\times d}$ is the embedding matrix computed using the different types of nodes mentioned in the previous section.

After the representation learning process, the model should be able to better identify and extract features under different augmentation strategies. However, random augmentation strategies cannot guarantee the stability of the original graph after perturbation and may integrate non-sensitive information into the embedding of the original nodes. We want the model to accurately extract sensitive information even when non-sensitive information changes. This process can be represented by Equation 5.

(Equation 5) $$\begin{array}{c} p\left(Y\left|S_{s},S_{n}=S_{ni})=p(Y\right|S_{s},S_{n}=S_{n*}\right) \end{array}$$

where S_n_=S_ni_ and S_n_=S_n∗_ indicates that when the information of non-sensitive nodes changes, the model focuses more on extracting information from the sensitive nodes S_s_.

Therefore, we adjust the model’s objective function to focus on sensitive node variables, allowing perturbations in non-sensitive information while maintaining the stability of sensitive information. We also employ Multi-head Graph Attention Networks to aggregate neighborhood information, integrating node features and structural characteristics to achieve consistent representation learning in augmented graphs. As shown as (Equation 6), (Equation 7), (Equation 8), the final embedding comprehensively represent the feature of augmented graph is follow:

(Equation 6) $$\begin{array}{c} T_{i}^{l-1}={∥}_{k=1}^{K}\sigma \left(\sum_{j\in N_{j}}\alpha_{ij}^{k}W^{k}T_{j}\right) \end{array}$$

(Equation 7) $$\begin{array}{c} T_{i}^{l}=\sigma \left(\frac{1}{K}\sum_{k=1}^{K}\sum_{j\in N_{j}}\alpha_{ij}^{k}W^{k}T_{j}\right) \end{array}$$

(Equation 8) $$\begin{array}{c} X_{i}=Meanpolling\left(\sum_{i\in G}T_{i}H_{i}\right) \end{array}$$

where $T_{i}^{l-1}$ represents the concatenation of the results from each head in the first l-1 layers, and ‖ denotes the concatenation operation, and K represents the number of multi-heads. $T_{i}^{l}$ represents the last layer, where the K attention results are averaged. α_ij_ = softmax(e_ij_) is the attention score and e_ij_ = LeakyReLU(a[WT_i_∥WT_j_]), and a is a learnable parameter used to compute the attention weights.

Finally, the original graph G_ori_=(A,H) shares the parameters of the GAT with the augmented graph, using it to learn the feature representations of the original graph.

Based on the above operations, we can obtain the embedding matrix of the two augmented graphs X_1_ and X_2_. As previous studies have pointed out (Qiao et al. 2024), when regularizing the embedding representations of X_1_ and X_2_, ensuring that X_1_ and X_2_ have the same mean and variance can guarantee that the embedding of the same augmented graphs obtained in different ways remain stable. This learning objective can be formulated as Equation 9:

(Equation 9) $$\begin{array}{c} \left\{ \begin{array}{c} \min\sum {\left(∥X_{1}-X_{2}∥\right)}^{2}\\ Std\left(X_{1}\right)=Std\left(X_{2}\right)∼\varphi \end{array}\right. \end{array}$$

where X_1_ and X_2_ represent the two embedding matrix, and Std(·) is used to calculate the standard deviation. The first part ensures that the means of the two embedding matrix are the same, while the second part aims to make the standard deviation close to φ.

To ensure that the embedding have the same mean, the Euclidean distance between the embedding matrices can be used as an indicator. To make the standard deviation closer to φ, we minimize ∥Std(X_i_)-φ∥^2^. The proposed augmented graph loss function is as Equation 10:

(Equation 10) $$\begin{array}{c} L_{1}=\omega_{1}∥X_{1}^{i}-X_{2}^{i}∥+\omega_{2}\sum_{i}\left(\sqrt{∥s_{1}^{i}-\varphi {∥}^{2}}+\sqrt{∥s_{2}^{i}-\varphi {∥}^{2}}\right) \end{array}$$

where ω_1_ and ω_2_ are weight parameters used to balance different parts, s_1_ and s_2_ respectively denote the standard deviation of the embedding matrices of X_1_ and X_2_, φ is the standard deviation of the embedding matrix, determined by the average standard deviation of cell line or drug embeddings.

For the other part, we define drug response prediction as a binary classification task. As shown as Equation 11, we use the focal loss function between the predicted results and the true value labels:

(Equation 11) $$\begin{array}{c} L_{2}=-\alpha_{t}{\left(1-p_{t}\right)}^{\gamma }\;\log\left(p_{t}\right) \end{array}$$

where α_t_ is the focusing parameter, which controls the degree of suppression on easy-to-classify samples; γ is the balance factor, which adjusts the weights of positive and negative samples and p_t_ is the model’s predicted probability for the true category.

Finally, the optimization objective of the model (Equation 12) consists of the two parts mentioned above.

(Equation 12) $$\begin{array}{c} L=L_{1}+L_{2} \end{array}$$

Existing graph augmentation methods apply uniform perturbation to all nodes, which may destroy useful structural information. MOGA’s type-specific perturbation, only non-sensitive nodes are masked preserves critical interactions between sensitive nodes, for example, if cell line A sensitive to drug B is a sensitive node pair, their edge is never masked, ensuring the model retains core biological signals.

### Quantification and statistical analysis

Evaluation metrics include AUC, AUPR, accuracy, precision, recall, and F1 score for binary classification. These performance metrics reflect the model’s performance on test instances, not on biologically replicated samples. Our main results are presented as mean ± standard error.
