# Supplementary material for: The MOGA multi-modal framework based on graph augmentation networks for drug response prediction
Source: iScience. 2026 Mar 11;29(4):115167. doi: 10.1016/j.isci.2026.115167 (PMC13092757; doi:10.1016/j.isci.2026.115167)
Supplement: Document S1. Tables S1 and S2 and Data S1 [file mmc1.pdf]

iScience, Volume *29*

Supplemental information

The MOGA multi-modal framework based on graph augmentation networks for drug response prediction

Kaiyuan Zhang, Runze Wang, Tianyi Zang, and Yanli Zhao

Table S1: Multi-Omics Convolution Layers, related to METHOD DETAILS of STAR METHODS.

**Omics Type**	**Original Dimension**	**Convolution Structure**	**Output Dimension**
Transcriptomics	16,382	1×50Convolution(128 kernels) → ReLU → BatchNorm → Dropout(0.3) → 1×20Convolution(128 kernels) → ReLU → BatchNorm → Max Pooling → Fully Connected (256D)	256
Proteomics	12,755	1×30Convolution(64 kernels) → ReLU → BatchNorm → Dropout(0.2) → 1×15Convolution(64 kernels) → ReLU → BatchNorm → Max Pooling → Fully Connected (256D)	256
Copy Number Variation	16,382	1×50Convolution(128 kernels) → ReLU → BatchNorm → Dropout(0.3) → 1×20Convolution(128 kernels) → ReLU → BatchNorm → Max Pooling → Fully Connected (256D)	256
Mutations	16,381	1×50Convolution(128 kernels) → ReLU → BatchNorm → Dropout(0.3) → 1×20Convolution(128 kernels) → ReLU → BatchNorm → Max Pooling → Fully Connected (256D)	256
DNA Methylation	14,234	1×50Convolution(64 kernels) → ReLU → BatchNorm → Dropout(0.2) → 1×20Convolution(64 kernels) → ReLU → BatchNorm → Max Pooling → Fully Connected (256D)	256
Metabolomics	225	1×10Convolution(32 kernels) → ReLU → BatchNorm → Dropout(0.2) → 1×5Convolution(32 kernels) → ReLU → BatchNorm → Max Pooling → Fully Connected (256D)	256

Table S2. Computational costs, related to Augmentation Graph Analysis (RQ3) of Results.

	Training	Inference(single-sample)
Origin dataset	2.2h	1.6s
Extended dataset	15.6h	3.2s

Data S1: Scalability Strategy for Large-Scale Datasets, related to Augmentation Graph Analysis (RQ3) of Results

To adapt to ultra-large-scale datasets, we propose three core optimization schemes:

(1) Mini-Batch Training Based on Node Sampling

We adopt GraphSAGE-style neighborhood sampling (20 neighbors sampled per node) to reduce the computational load per iteration. For datasets with E=108, the number of effective edges per iteration can be reduced to 2×106, cutting training time by 80%.

(2) Model Distillation

Using a pre-trained large model as the teacher, we train a lightweight student model. The student model retains 98% of the teacher model’s performance while reducing the parameter scale .

(3) Parallel Computing and Hardware Acceleration

Multi-GPU parallelism: Assign multi-omics integration and drug encoding to 4 GPUs.
